# XDR-TB Transmitted from Mother to 10-Month-Old Infant: Diagnostic and Therapeutic Problems

**DOI:** 10.3390/diagnostics12020438

**Published:** 2022-02-08

**Authors:** Monika Kozińska, Krystyna Bogucka, Krzysztof Kędziora, Jolanta Szpak-Szpakowska, Wiesława Pędzierska-Olizarowicz, Andrzej Pustkowski, Ewa Augustynowicz-Kopeć

**Affiliations:** 1 Department of Microbiology, National Tuberculosis and Lung Diseases Research Institute, Plocka 26, 01-138 Warsaw, Poland; e.kopec@igichp.edu.pl; 2 Medical Laboratory BRUSS, ALAB Group, Department of Mycobacterium Tuberculosis Diagnostics, Powstania Styczniowego 9B, 81-519 Gdynia, Poland; krystyna.bogucka@lmbruss.pl; 3 Department of Tuberculosis and Lung Diseases, Specialist Hospital in Prabuty, Kuracyjna 30, 82-550 Prabuty, Poland; kkedz@gumed.edu.pl (K.K.); jolantaszpak@op.pl (J.S.-S.); 4 Department of Allergology, Immunology and Lung Diseases, The Maciej Płażyński Polanki Children’s Hospital, Polanki 119, 80-308 Gdansk, Poland; w.pedzierska@szpitalpolanki.pl; 5 Department of Tuberculosis and Lung Diseases, Hospital Specialist Clinic Polanki, Polanki 119, 80-308 Gdansk, Poland; a.pustkowski@szpitalpolanki.pl

**Keywords:** *Mycobacterium tuberculosis*, drug resistance, XDR-tuberculosis, household TB transmission

## Abstract

Drug-resistant TB (DR-TB) in children is a special epidemiological, clinical, and diagnostic problem, and its global incidence remains unknown. DR-TB in children is usually of a primary nature and is most often transmitted to the child from a household contact, so these cases reflect the prevalence of DR-TB in the population of adult patients. The risk of infection with *Mycobacterium tuberculosis* complex (MTBC) in children depends on age, duration of exposure, proximity of contact with the infected person, and the level of source virulence. Most cases of TB in children, especially in infants, are caused by household contacts, where the main sources of infection are parents, grandparents or older siblings. However, there are many documented cases of TB transmission outside the family. The most common source of infection is an adult who is profusely positive for mycobacteria, diagnosed too late, and inadequately treated. It has been estimated that a sputum-positive patient might infect 30–50% of their household members. For this reason, active epidemiological investigation and contact tracing in the environment of sputum-positive patients are the most appropriate methods of identifying infected family members. This paper presents a case report concerning the transmission of extensively drug-resistant TB, Beijing 265 genotype, from a mother to her 10-month-old daughter. It is the first case diagnosed in Poland, and one of very few described in the literature where treatment was effective in the mother and the infant recovered spontaneously.

## 1. Introduction

Globally, about one million children are diagnosed with tuberculosis (TB) each year, and 210,000 die because of TB-related complications [1]. The accurate estimation of the global burden of childhood tuberculosis is difficult, mainly due to the problems associated with the detection, diagnosis, and insufficient surveillance of the disease, especially in countries with high TB-incidence rates [2]. According to WHO estimates, children younger than 15 years account for 15–20% of the global TB burden, but the number of reported cases varies greatly between regions and ranges from 3 to 25% [3,4].

In Poland, childhood tuberculosis is not an epidemiologically significant problem, possibly due to low TB incidence in the general population. In 2018 and 2019, children accounted for 0.9% and 1.5% of all reported TB cases, respectively. The number of notified cases was 20 (38%) vs. 26 (32%) for the age group 0–4 years, and 32 (62%) vs. 55 (68%) for the age group 5–14 years, in 2018 and 2019, respectively [5].

Drug-resistant TB (DR-TB) in children is a special epidemiological, clinical, and diagnostic problem, and its global incidence remains unknown. The latest data indicate that around 25,000 to 32,000 children worldwide develop DR-TB annually, which accounts for 3% of all childhood tuberculosis cases [6].

DR-TB in children is usually of a primary nature and is most often transmitted to the child from a household contact, so these cases reflect the prevalence of DR-TB in the population of adult patients [7].

TB in children is often paucibacillary and therefore difficult to confirm using microbiological methods. Therefore, the diagnosis is often presumptive and based on a medical interview. It is believed that a child diagnosed based on clinical symptoms, who recently had close contact with an MDR-TB (resistant to at least isoniazid and rifampin) patient, should be treated according to the drug-susceptibility test performed for the patient identified as the likely source of infection. It was confirmed that the concordance of the drug-resistance profile of strains isolated from children aged <15 years and the source patient may reach the level of 96% [8].

The risk of infection with *Mycobacterium tuberculosis* complex (MTBC) in children depends on the age, duration of exposure, proximity of contact with the infected person, and the level of source virulence. Most cases of TB in children, especially in infants, are caused by household contacts, where the main sources of infection are parents, grandparents or older siblings. However, there are many documented cases of TB transmission outside the family, such as kindergartens, schools, family nursing homes, churches, school buses, and shops [9,10]. The most common source of infection is an adult who is profusely positive for *Mycobacterium tuberculosis*, diagnosed too late, and inadequately treated. It has been estimated that a sputum-positive patient might infect 30–50% of their household members. For this reason, active epidemiological investigation and contact tracing in the environment of sputum-positive patients are the most appropriate methods of identifying infected family members [11].

The present paper presents a case of transmission of extensively drug-resistant TB (XDR-TB, resistant to isoniazid and rifampin, plus any fluoroquinolone, and at least one of three injectable second-line drugs), Beijing 265 genotype, from a mother to her 10-month-old daughter. It is the first case in Poland and one of very few described in the world where treatment was effective in the mother and the infant recovered spontaneously.

## 2. Case Study

### 2.1. Mother, Age 27 Years

**Figure 1:**
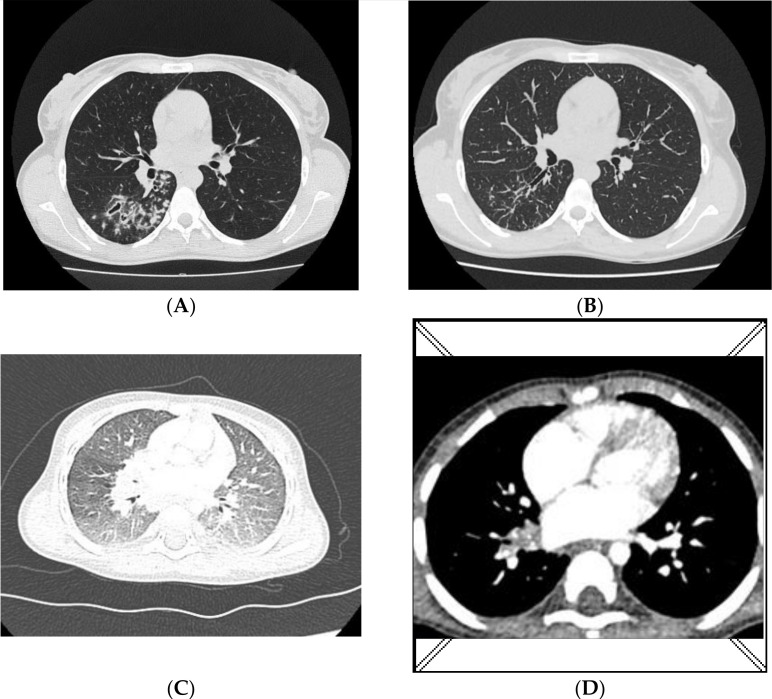
Chest CT **A/B**—mother; (**A**)—nodular lesions in the right lung (3 June 2020); (**B**)—regression of lesions (5 November 2020); **C/D**—infant; (**C**)—ground-glass opacities with some consolidations localized in both lungs (12 June 2020); (**D**)—regression of lesions, numerous calcifications in the nodes of the right hilum and subcarinal nodes (5 October 2020).

In January 2020, a 27-year-old woman with symptoms of tuberculosis visited a pulmonology clinic. For 2 months, she had a cough with mild haemoptysis and fever. A sputum smear was positive for mycobacteria (Acid Fast Bacilli, AFB+++), and the treatment was initiated according to the following regimen: rifampicin, isoniazid, ethambutol, and pyrazinamide. No improvement was achieved after 2 months of therapy, and an X-ray of the lungs on 24 March 2020 revealed patchy and nodular infiltrations localized in the right lung. The strain isolated from the sputum was identified as XDR-MTB, spoligotype Beijing 265. All molecular and biochemical tests were performed in the National Tuberculosis and Lung Diseases Research Institute in Warsaw, Poland. According to the drug-resistance phenotype determined on the liquid medium, treatment was introduced for drugs to which the strain was sensitive: linezolid, cycloserine, ethambutol, levofloxacin and ethionamide. The patient was still profusely positive for mycobacteria (AFB++). A bronchial secretion was collected on 1 April 2020 and the XDR strain, Beijing 265 genotype, was cultured again. Treatment was continued, but levofloxacin was substituted with moxifloxacin. A chest CT performed on 3 June 2020 revealed the presence of diffuse nodular lesions localized in the right lung (Figure 1A). Therapy with ethambutol was discontinued due to ophthalmic problems, while linezolid and ethionamide were discontinued because of sensory polyneuropathy. Follow-up tests for AFB (culture in solid and liquid media and detection of DNA directly in the clinical specimen) were negative in June 2020. After 6 months of antibiotic treatment, a CT scan revealed the partial regression of lesions (Figure 1B). The patient’s clinical status improved and treatment with bedaquiline, moxifloxacin, and cycloserine was finally sustained.

### 2.2. Daughter, Age 10 Months

The infant was first examined at the pulmonology outpatient clinic at the beginning of February 2020 when the mother tested positive for mycobacteria in the sputum (AFB+++), and antimycobacterial treatment was initiated, with continued microbiological tests for TB (inoculation on solid and liquid media and an antibiogram). A chest X-ray of the infant did not reveal any lesions in the lungs, and the IGRA (interferon-gamma release assay) was negative. A 3-month prophylactic treatment with isoniazid was initiated. In May 2020, the IGRA was repeated, and the result was positive. The infant was in good health without clinical symptoms. Gastric lavage specimens were sampled three times for microbiological tests. In June 2020, a chest X-ray and CT scan were performed again. Ground-glass opacities were identified in the lungs as well as enlarged lymph nodes in the lung hila and mediastinum (Figure 1C). A bronchoscopy was also performed, and the mucous secretion was aspirated and sent for microbiological tests. Because the infant was in good physical health and had no clinical symptoms, and the fact that the strain isolated from the mother was multi-drug resistant (no guidelines for prophylaxis in children having contact with XDR-TB), treatment was not initiated, and a decision was made to conduct weekly follow-up tests at the pulmonology outpatient clinic.

In July 2020, an MTBC strain was cultured from gastric lavage specimens sampled in May. The cultured strain was identical to the one cultured from the mother: XDR-TB, spoligotype Beijing 265 with MIRU-VNTR code 333654444432658 (Table 1). In October 2020, after a 3-month observation, gastric lavage specimens were collected three times again. Microbiological tests were negative. A CT was also repeated. Compared to the image acquired in June 2020, regression of the lesions was observed (Figure 1D), the lymph nodes decreased in size, and numerous minor calcifications appeared.

Because the infant still had no clinical symptoms, antimycobacterial treatment was not initiated, and follow-up in the outpatient setting was continued. The girl remains in good health to this day, and her mental and physical development is normal.

In the reported household, there were also two other children, aged two and four years, in whom latent infection with *Mycobacterium tuberculosis* was confirmed using QuantiFERON-TB Gold Plus (QFT-Plus; Qiagen, Hilden, Germany).

## 3. Discussion

The described case concerns the transmission of Beijing type XDR-TB between family members in one household, where the source of infection was the mother, who was profusely positive for mycobacteria. TB in the 10-month-old daughter was confirmed in a microbiological test by isolating the XDR strain of the Beijing 265 genotype from gastric lavage, and it had an identical molecular code to the strain isolated from the mother. Due to the absence of clinical symptoms of TB in the infant, antimycobacterial treatment was not initiated, and the child was followed-up in the outpatient setting.

Cases of tuberculosis in children are reported many times, and a claim has been made that they are able to control the disease progression with no need for clinical intervention. Research from the period before the use of chemotherapy shows that the majority of children recover from tuberculosis without any treatment, and pathological changes in the lungs seen in radiographs often resolve spontaneously. It has also been demonstrated that *Mycobacterium tuberculosis* strains can be cultured from recently infected asymptomatic children [12,13,14]. Contemporary studies, including the described case, provide information on TB in children with microbiologically confirmed asymptomatic MDR-TB that requires no treatment [15,16,17].

There may be various reasons for the presence of MTBC in biological specimens collected from the respiratory tract of asymptomatic children. First, it can result from the natural history of infection where children, after a recent primary infection, might temporarily shed viable mycobacteria in the absence of an active form of the disease. This phenomenon was first documented in the 1930s [16]. Second, latent infection covers the spectrum of clinical situations, from inactive (latent) mycobacterial infection to periods of subclinical proliferation of *Mycobacterium tuberculosis*, which, however, is insufficient to cause lung damage [18,19].

How, then, should we classify a totally asymptomatic person with microbiologically confirmed tuberculosis but without clinical or radiological symptoms suggesting an active disease? Is it a case of an active disease?

Tuberculosis in children, especially XDR-TB, is still a serious therapeutic problem. Guidelines on its treatment are similar to those that apply to adults, but there are no antimycobacterial medications dedicated to the youngest population of patients [20]. The only dosage regimen relies on dividing a tablet normally prescribed to adults, which is highly controversial. In 2017, paediatric TB physician Dr Jeffery Starke in his speech opening the 48^th^ World Union Conference on Lung Health emphasized that “(…) children have the same right as adults to benefit from tuberculosis care and research. It is time that we put these words into action (…)” [21].

Although drugs for MDR-TB in adults are used in older children, currently there are no recommendations on the administration of bedaquiline to children aged <6 years (<15 kg bw) due to the lack of data on its pharmacokinetics and safety [22]. In the treatment of XDR-TB in children aged 3–6 years, bedaquiline can be replaced with delamanide, but it should not be used in children <3 years (<10 kg bw) [23].

The number of XDR-TB cases in young children reported to date is limited. There are fragmentary data on the treatment of XDR-TB in children, ending either with success or fatality [24].

In the described household, apart from the 10-month-old infant, there were also two other infected young children, but they have not developed active tuberculosis so far. It is known that not all infected people have the same risk of developing the active form of TB. In the immunocompetent adult population, the lifetime risk of TB is approximately 5 to 10%; half of these cases develop active TB in the first 2–3 years following infection. On the other hand, in the population of immunocompetent infected infants who received no prophylactic treatment, up to 50% develop active TB within 6–9 months after infection, and the disease might be severe and life threatening [1]. International guidelines recommend monitoring close contacts of patients with MDR-TB for at least 2 years, or for at least 4 years in the case of XDR-TB [25].

It has been proven that BCG vaccination is important in the course of tuberculosis in children. The protective effect of the vaccine is manifested in the form of a lower incidence of tuberculosis in vaccinated children and less frequent progression of the latent form into active disease. Despite the fact that BCG vaccination does not eliminate the risk of infection among children in close contact with smear-positive adults, it significantly influences the course of the disease, protecting against severe TB, especially in the youngest children [26].

Particular attention should be paid to the fact that the described case concerns the transmission of MDR-TB caused by the Beijing genotype *Mycobacterium tuberculosis*. In antimycobacterial therapy, the choice of medication is based on drug-susceptibility testing, but in the case of Beijing-TB, the problem appears to be more complex. Some studies have suggested that exposure to Beijing strains is more often associated with progression to the active form of TB than in the case of infection with mycobacteria, representing other genotypes [27]. However, Canadian studies showed no such correlation [28]. Analyses carried out in Vietnam and Iran proved that Beijing-TB is more common in the population of younger adults, and the incidence of this disease decreases with age [29,30].

Undoubtedly, the Beijing genotype is associated with an increased risk of acquired drug resistance and a more difficult clinical course. The increased transmissibility of “Beijing strains” compared to other molecular families of MTBC has also been confirmed. In the present case report, we described the transmission of a strain identified as the Beijing 265 subtype. In contrast to the Beijing 1 subtype, detected in patients with a spectrum of TB forms, from drug sensitive to XDR-TB, the Beijing 265 clone in Poland has been isolated only from patients with MDR, pre-XDR and XDR-TB [31].

## 4. Conclusions

This paper addresses the important social and epidemiological problems of contact tracing between family members and the active detection of tuberculosis infection sources in households with young children. It is known that such children are exposed to a small number of potential sources of infection, and the time of potential TB transmission is limited. Yet, the level of source-case detection in many populations worldwide remains low, implying a missed opportunity in TB eradication. The morbidity and mortality of tuberculosis in children reflect the quality of surveillance of transmission from adults. The effective control of this infectious disease requires, first of all, a careful estimate of morbidity and mortality in children. Professional medical care should be provided not only to paediatric patients diagnosed with tuberculosis, but also their close family.

The described case implies that currently TB control programs should prioritize the development and implementation of guidelines on chemoprophylaxis in patients with XDR-TB, especially in young children infected after contact with their family members.

## Figures and Tables

**Table 1:** Results of microbiological and genetic assays for MTBC strains cultured from the mother and infant.

	Mother		Daugther
Clinical material	Sputum	Bronchial secretion	Gastric lavage
Date of strain isolation	January 2020	April 2020	July 2020
Species	*Mycobacterium tuberculosis*		*Mycobacterium tuberculosis*
Drug resistance profile	XDR Resistance to isoniazid, rimfampicin, ofloxacin, amikacin, kanamycin, capreomycin		XDR Resistance to isoniazid, rimfampicin, ofloxacin, amikacin, kanamycin, capreomycin
Spoligotype	Beijing 265		Beijing 265
MIRU-VNTR code	333654444432658		333654444432658

## Data Availability

The results of the presented research are archived in the documentation of hospitals where mother and daughter were treated: Department of Tuberculosis and Lung Diseases, Specialist Hospital in Prabuty, Kuracyjna 30, 82-550 Prabuty, Poland and Department of Allergology, Immunology and Lung Diseases, The Maciej Płażyński Polanki Children’s Hospital, Polanki 119, 80-308 Gdansk, Poland. Microbiological documentation has been archived at the Department of Microbiology, National Tuberculosis and Lung Diseases Research Institute, Plocka 26, 01-138 Warsaw, Poland.

## References

[1] Lamb G.S., Starke J.R. (2017). Tuberculosis in Infants and Children. Microbiol. Spectr..

[2] Newton S.M., Brent A.J., Anderson S., Whittaker E., Kampmann B. (2008). Paediatric tuberculosis. Lancet Infect. Dis..

[3] Marais B.J., Gupta A., Starke J.R., El Sony A. (2010). Tuberculosis in women and children. Lancet.

[4] World Health Oganization (2014). Guidance for National Tuberculosis Programmes on the Management of Tuberculosis in Children.

[5] Korzeniewska-Koseła M. (2020). Tuberculosis and Lung Diseases in Poland in 2019.

[6] Tola H.H., Holakouie-Naieni K., Mansournia M.A., Yaseri M., Tesfaye E., Mahamed Z., Molla Sisay M. (2020). Low enrollment and high treatment success in children with drug-resistant tuberculosis in Ethiopia: A ten years national retrospective cohort study. PLoS ONE.

[7] Schaaf H.S., Marais B.J., Hesseling A.C., Brittle W., Donald P.R. (2009). Surveillance of antituberculosis drug resistance among children from the Western Cape Province of South Africa—An upward trend. Am. J. Public Health.

[8] Steiner P., Rao M., Mitchell M., Steiner M. (1985). Primary drug-resistant tuberculosis in children. Correlation of drug-susceptibility patterns of matched patient and source case strains of Mycobacterium tuberculosis. Am. J. Dis. Child..

[9] Kozińska M., Augustynowicz-Kopeć E. (2016). The incidence of tuberculosis transmission among family members and outside households. Pneumonol. Alergol. Pol..

[10] Augustynowicz-Kopeć E., Jagielski T., Kozińska M., Kremer K., van Soolingen D., Bielecki J., Zwolska Z. (2012). Transmission of tuberculosis within family-households. J. Infect..

[11] Ho J., Fox G.J., Marais B.J. (2016). Passive case finding for tuberculosis is not enough. Int. J. Mycobacteriol..

[12] Jenkins H.E., Yuen C.M., Rodriguez C.A., Nathavitharana R.R., McLaughlin M.M., Donald P., Marais B.J., Becerra M.C. (2017). Mortality in children diagnosed with tuberculosis: A systematic review and meta-analysis. Lancet Infect. Dis..

[13] Donald P.R., Marais B.J., Barry C.E. (2010). Age and the epidemiology and pathogenesis of tuberculosis. Lancet.

[14] Marais B.J., Gie R.P., Schaaf H.S., Hesseling A.C., Obihara C.C., Starke J.J., Enarson D.A., Donald P.R., Beyers N. (2004). The natural history of childhood intra–thoracic tuberculosis: A critical review of literature from the pre-chemotherapy era. Int. J. Tuber. Lung. Dis..

[15] Wallgren A. (1935). Primary pulmonary tuberculosis in childhood. Am. J. Dis. Child..

[16] Loveday M., Sunkari B., Marais B.J., Master I., Brust J.C. (2016). Dilemma of managing asymptomatic children referred with ‘culture-confirmed’ drug-resistant tuberculosis. Arch. Dis. Child..

[17] Seddon J., Godfrey-Faussett P., Hesseling A., Gie R.P., Beyers N., Schaaf H.S. (2012). Management of children exposed to multidrug-resistant Mycobacterium tuberculosis. Lancet Infect. Dis..

[18] Barry C.E., Boshoff H.I., Dartois V., Dick T., Ehrt S., Flynn J., Schnappinger D., Wilkinson R.J., Young D. (2009). The spectrum of latent tuberculosis: Rethinking the biology and intervention strategies. Nat. Rev. Microbiol..

[19] Cardona P.J. (2010). Revisiting the natural history of tuberculosis. The inclusion of constant reinfection, host tolerance, and damage-response frameworks leads to a better understanding of latent infection and its evolution towards active disease. Arch. Immunol. Ther. Exp..

[20] Seddon J.A., Schaaf H.S., Marais B.J., McKenna L., Garcia-Prats A.J., Hesseling A.C., Hughes J., Howell P., Detjen A., Amanullah F. (2018). Time to act on injectable-free regimens for children with multidrug-resistant tuberculosis. Lancet Respir. Med..

[21] Migliori G.B., Tiberi S., Zumla A., Petersen E., Chakaya J.M., Wejse C., Muñoz Torrico M., Duarte R., Alffenaar J.W., Schaaf H.S. (2020). Members of the Global Tuberculosis Network. MDR/XDR-TB management of patients and contacts: Challenges facing the new decade. The 2020 clinical update by the Global Tuberculosis Network. Int. J. Infect. Dis..

[22] The Union WHO Global TB Symposium. Proceedings of the 48th Union World Conference on Lung Health.

[23] Huynh J., Marais B.J. (2019). Multidrug-resistant tuberculosis infection and disease in children: A review of new and repurposed drugs. Ther. Adv. Infect. Dis..

[24] Katragkou A., Antachopoulos C., Hatziagorou E., Sdougka M., Roilides E., Tsanakas J. (2013). Drug-resistant tuberculosis in two children in Greece: Report of the first extensively drug-resistant case. Eur. J. Pediatr..

[25] Becerra M.C., Appleton S.C., Franke M.F., Chalco K., Arteaga F., Bayona J., Murray M., Atwood S.S., Mitnick C.D. (2011). Tuberculosis burden in households of patients with multidrug-resistant and extensively drug-resistant tuberculosis: A retrospective cohort study. Lancet.

[26] Soysal A., Millington K.A., Bakir B., Dosanjh D., Aslan Y., Deeks J.J., Efe S., Staveley I., Ewer K., Lalvani A. (2005). Effect of BCG vaccination on risk of Mycobacterium tuberculosis infection in children with household tuberculosis contact: A prospective community-based study. Lancet.

[27] Hanekom M., van der Spuy G.D., Streicher E., Ndabambi S.L., McEvoy C.R., Kidd M., Beyers N., Victor T.C., van Helden P.D., Warren R.M. (2007). A recently evolved sublineage of the Mycobacterium tuberculosis Beijing strain family is associated with an increased ability to spread and cause disease. J. Clin. Microbiol..

[28] Langlois-Klassen D., Senthilselvan A., Chui L., Kunimoto D., Saunders L.D., Menzies D., Long R. (2013). Transmission of Mycobacterium tuberculosis Beijing Strains, Alberta, Canada, 1991–2007. Emerg. Infect. Dis..

[29] Buu T.N., Huyen M.N., Lan N.T., Quy H.T., Hen N.V., Zignol M., Borgdorff M.W., Cobelens F.G., van Soolingen D. (2009). The Beijing genotype is associated with young age and multidrug-resistant tuberculosis in rural Vietnam. Int. J. Tuberc. Lung. Dis..

[30] Erie H., Kaboosi H., Javid N., Shirzad-Aski H., Taziki M., Kuchaksaraee M.B., Ghaemi E.A. (2017). The high prevalence of Mycobacterium tuberculosis Beijing strain at an early age and extra-pulmonary tuberculosis cases. Iran J. Microbiol..

[31] Kozińska M., Augustynowicz-Kopeć E. (2015). Drug Resistance and Population Structure of Mycobacterium tuberculosis Beijing Strains Isolated in Poland. Pol. J. Microbiol..

